# Psychiatric morbidity during the multiple sclerosis prodrome is associated with future disability

**DOI:** 10.1177/13524585251382801

**Published:** 2025-10-26

**Authors:** Anibal S Chertcoff, Marta Ruiz-Algueró, Fardowsa Yusuf, Yinshan Zhao, Feng Zhu, Ruth Ann Marrie, Helen Tremlett

**Affiliations:** Department of Internal Medicine (Neurology), Health Sciences Centre, Max Rady College of Medicine, Rady Faculty of Health Sciences, University of Manitoba, Winnipeg, MB, Canada; Faculty of Medicine (Neurology), University of British Columbia and The Djavad Mowafaghian Centre for Brain Health, Vancouver, BC, Canada; Faculty of Medicine (Neurology), University of British Columbia and The Djavad Mowafaghian Centre for Brain Health, Vancouver, BC, Canada; School of Population and Public Health, University of British Columbia, Vancouver, BC, Canada; Faculty of Medicine (Neurology), University of British Columbia and The Djavad Mowafaghian Centre for Brain Health, Vancouver, BC, Canada; Faculty of Medicine (Neurology), University of British Columbia and The Djavad Mowafaghian Centre for Brain Health, Vancouver, BC, Canada; Department of Medicine, Faculty of Medicine, Dalhousie University, Nova Scotia Health, Halifax, NS, Canada; Faculty of Medicine (Neurology), University of British Columbia and The Djavad Mowafaghian Centre for Brain Health, Vancouver, BC, Canada

**Keywords:** Multiple sclerosis, prodrome, psychiatric morbidity, neurological disability, population-based data

## Abstract

**Background:**

Evidence suggests a prodromal phase in multiple sclerosis (MS) identifiable via healthcare use, including psychiatric symptoms. The association between psychiatric morbidity in this phase and future outcomes remains unclear.

**Objectives:**

We investigated the association between psychiatric morbidity in the 5-years pre-MS onset and subsequent disability (EDSS) scores.

**Methods:**

We identified MS patients who visited an MS clinic in British Columbia, Canada (1991–2018) and linked their clinical and population-based health administrative data. Psychiatric morbidity was identified using physician/hospital visits in the 5-years pre-MS onset. Multivariable generalized linear models examined the association between psychiatric morbidity and subsequent EDSS scores. We explored effect modification by sex, age, and MS course and investigated if high psychiatric-related physician visits (>median) or hospitalizations (measures of “psychiatric morbidity burden”) were associated with EDSS scores.

**Results:**

Among 2212 MS patients, 481 (21.7%) had psychiatric morbidity in the 5-years pre-MS onset. Follow-up averaged 5.2 (SD: 4.9) years (first-to-last EDSS assessment). Psychiatric morbidity pre-MS onset was associated with higher post-diagnosis EDSS scores (covariate-adjusted[adj) β = 0.17; 95% confidence interval (CI): 0.03–0.30). Associations were more pronounced in males (adjβ = 0.43; 95% CI: 0.04–0.83), <30 years (adjβ = 0.44; 95% CI: 0.15–0.73), relapsing-onset-MS (adjβ = 0.22; 95% CI: 0.08–0.37) and high psychiatric-related physician visit burden (adjβ = 0.23; 95% CI: 0.05–0.41), or hospitalizations (adjβ = 0.48; 95% CI: 0.002–0.96).

**Conclusions:**

Psychiatric morbidity before MS recognition was associated with increased future disability, particularly in males, younger individuals, relapsing-onset-MS, and high pre-MS onset psychiatric morbidity burden.

## Introduction

Growing evidence has indicated the presence of a potential prodromal phase in multiple sclerosis (MS).^1^ A psychiatric dimension to this prodrome has also emerged.^2^ In the 5 years before disease onset, people with MS (pwMS) are more likely to experience psychiatric conditions compared with the general population,^3^ and to use more mental health services including physician visits, and hospital admissions.^4^

Psychiatric disease negatively affects quality of life of pwMS.^5^ Its presence after classical MS symptom onset is also associated with greater disability accumulation over time.^6^ However, our understanding of the association between psychiatric morbidity occurring before the formal recognition of MS and subsequent disability accrual remains limited.

Utilizing linked clinical and health administrative data, we explored the relationship between psychiatric morbidity in the 5 years before MS symptom onset (the presumed prodromal period) and subsequent neurological disability, measured by the Expanded Disability Status Scale (EDSS), to investigate the link between early psychiatric morbidity and disability accumulation in MS. In addition, we evaluated how the frequency of healthcare utilization for psychiatric morbidity (i.e., number of physician visits and hospitalizations) in the 5-years pre-MS symptom onset, as a measure of psychiatric morbidity burden, is associated with EDSS in pwMS.

## Methods

### Data collection

This retrospective cohort study linked MS-specific clinical records with population-based health administrative data from the province of British Columbia (BC), Canada. BC has a population of 5.3 million and a publicly funded healthcare system that provides universal access to medical services for all residents. MS-specific clinical data were available in BC, and included MS symptom onset date, disease course (relapsing or progressive onset), and EDSS scores recorded by an MS neurologist at routine clinic visits with corresponding dates. Utilizing each individual’s unique personal health care number, clinical data were linked to prospectively collected health administrative information. These data captured information on virtually every health encounter province-wide, including physician visits/hospitalizations, with diagnoses coded using the International Classification of Diseases (ICD) 9/10 system, complemented by BC-specific codes.^7^ In addition, data included every disease-modifying drug (DMD) prescription dispensation at outpatient/community pharmacies in BC, as well as demographic (sex/age), socioeconomic, residency details (postal codes), and death dates. Socioeconomic status (SES) estimates (quintiles) were derived using a Statistics Canada algorithm linking each individual’s postal code with their census-derived mean neighborhood-level income.^8^ All data were de-identified before analyses.

### Study participants

The study cohort comprised all individuals diagnosed with MS at one of the four BC MS clinics based on the prevailing criteria. The study index date was the MS symptom onset date, as recorded by a neurologist during a clinic visit. To select incident MS, patients were required to reside in BC for ⩾90% of the days each year in the 5-years pre-index date. Individuals were followed from 5 years before MS symptom onset to their last recorded EDSS before death, emigration (>90 consecutive days without registration in the mandatory provincial health plan), or 30 September 2018 (study end). All data were available from 1 January 1991 to 30 September 2018. To allow a 5-years follow-up pre-MS onset, the earliest possible index date was 1 January 1996.

### Exposure and outcome

Psychiatric morbidity was defined using a validated case definition, encompassing ICD codes for depression, anxiety, bipolar disorder, and/or schizophrenia. The definition required ⩾5 relevant psychiatric-related physician visits occurring on different days and/or ⩾1 psychiatric-related hospitalization (Supplemental Table 1) within a 5-years period.^4,9^ Visits (physician or hospital) could contribute to the case definition from the 5-years pre-index date onwards, with ⩾1 visit occurring within 5-years pre-MS symptom onset. Psychiatric morbidity during the prodromal period was considered present from the earliest physician visit/hospitalization onwards.

The study outcome was neurological disability, measured by the EDSS.

Supplemental Figure 1 shows the study timeline.

### Statistical analysis

We summarized the characteristics of the study cohort at the index date and the presence of psychiatric morbidity in the 5 years before MS onset. Associations between psychiatric morbidity during the 5-years pre-MS symptom onset (the exposure) and all available EDSS scores (the outcome) were examined using a multivariable generalized linear model with an identity link fitted by generalized estimating equations (GEE) with an exchangeable working correlation structure. All EDSS scores collected during follow-up were analyzed; the GEE model accounted for dependence of repeated observations within individuals. Results were adjusted (adj) for sex, age at MS symptom onset (continuous), calendar year (continuous), SES (quintiles; Q1–5; Q1 (most deprived) as reference), and disease course (relapsing-onset vs progressive-onset) at the index date. Time-varying covariates at each EDSS assessment included disease duration, prior DMD exposure (yes/no), and comorbidity score (using the Charlson Comorbidity Index (CCI), with ICD codes from hospital/physician visits in the previous 12 months (excluding hemiplegia/paraplegia to avoid misclassifying MS as a comorbidity), and categorized as 0, 1, or ⩾2). Interaction terms were used to assess whether sex, age at MS onset (<30, 30–49, and ⩾50 years), and MS course modified the association between psychiatric morbidity and EDSS scores, as these are known to influence MS progression and psychiatric comorbidities.^10–14^ Results were presented as adjβ-coefficients with 95% confidence intervals (CIs). As a post hoc analysis to determine whether higher disability in participants with psychiatric morbidity stemmed from worse baseline status or from faster progression, we used the primary GEE model with baseline EDSS entered as an additional covariate.

### Complementary analyses

We explored the association between psychiatric-related burden and EDSS scores. Among individuals fulfilling the criteria for psychiatric morbidity, we defined a *high physician visit burden* as having psychiatric-related physician visits above the median. We used visit frequency as a proxy for higher illness burden as population studies show frequent attenders have higher chronic-disease and psychiatric morbidity. Similarly, we assessed individuals with a *hospitalization burden*, defined as having ⩾1 psychiatric-related hospitalization. Using a similar approach as before, multivariable generalized linear models examined the association of these two groups separately with subsequent EDSS scores, with individuals not meeting the criteria for psychiatric morbidity being the reference group.

Analyses were conducted using R (v4.1.0; Vienna, Austria).

The study was approved by University of British Columbia’s Clinical Research Ethics Board (H20-03232).

## Results

The cohort comprised 2212 pwMS; 73.6% were female. The average (SD) age at MS onset = 37.8 (11.3) years. At the index date, 14.6% had a CCI score ⩾ 1, and 89.3% had relapsing-onset MS. During the 5-years pre-MS onset, 481 (21.7%) had psychiatric morbidity. Of these, 86.5% were female, with an average (SD) age at MS onset of 39.1 (10.4) years; 89.0% had relapsing-onset MS (Table 1).

**Table 1.** Characteristics of patients with multiple sclerosis (MS) from British Columbia, Canada at MS symptom onset.

Characteristics	Total population (*N* = 2212)	No psychiatric morbidity (*N* = 1731)	Psychiatric morbidity‡ (*N* = 481)
Females, *n* (%)	1627 (73.6)	1211 (70.0)	416 (86.5)
Age at MS symptom onset in years, mean (SD)	37.8 (11.3)	37.5 (11.5)	39.1 (10.4)
Age group, *n* (%)			
<30 years	605 (27.4)	503 (29.1)	102 (21.2)
30 to 49 years	1273 (57.5)	971 (56.1)	302 (62.8)
⩾50 years	334 (15.1)	257 (14.8)	77 (16.0)
Socioeconomic status, *n* (%)			
1 (most deprived)	355 (16.0)	269 (15.5)	86 (17.9)
2	376 (17.0)	292 (16.9)	84 (17.5)
3 or missing*	514 (23.2)	362 (20.9)	107 (22.2)
4	491 (22.2)	389 (22.5)	102 (21.2)
5 (most affluent)	476 (21.5)	381 (22.0)	95 (19.8)
Index year, *n* (%)			
1996–2004	1271 (57.5)	992 (57.3)	279 (58.0)
2005–2013	821 (37.2)	642 (37.1)	179 (37.2)
2014–2018	120 (5.4)	97 (5.6)	23 (4.8)
Charlson Comorbidity score, *n* (%) †			
0	1890 (85.4)	1509 (87.2)	381 (79.2)
1	246 (11.1)	171 (9.9)	75 (15.6)
⩾2	76 (3.4)	51 (2.9)	25 (5.2)
MS disease course, *n* (%)			
Relapsing-onset	1975 (89.3)	1547 (89.4)	428 (89.0)
Primary progressive	237 (10.7)	184 (10.6)	53 (11.0)

MS: Multiple sclerosis; SD: standard deviation; N/A: Not available. *Information was missing for 45 (2.0%) patients. Those with missing SES were assigned to quintile 3. †Comorbidity score was measured by using the Charlson Comorbidity Index (excluding hemiplegia/paraplegia) during the year pre-index date. ‡Psychiatric morbidity was considered present when an individual presented at least five relevant psychiatric-related physician visits and/or one psychiatric-related hospital admission within a 5-years period before MS onset. Visits could contribute from the 5th-year pre-MS symptom onset onwards, with ⩾1 visit occurring during this period. Psychiatric morbidity was considered present from the earliest visit onwards.

The mean (SD) time from MS symptom onset to the first recorded EDSS was 3.2 (3.3) years and from first-to-last EDSS was 5.2 (4.9) years. The mean (SD) number of EDSS assessments was 5.5 (4.4). The median (IQR) EDSS at first assessment was 2.0 (1.5) and 2.5 (2.5) at last follow-up. A total of 1117 (50.5%) individuals were exposed to any DMD during follow-up.

**Figure 1.**
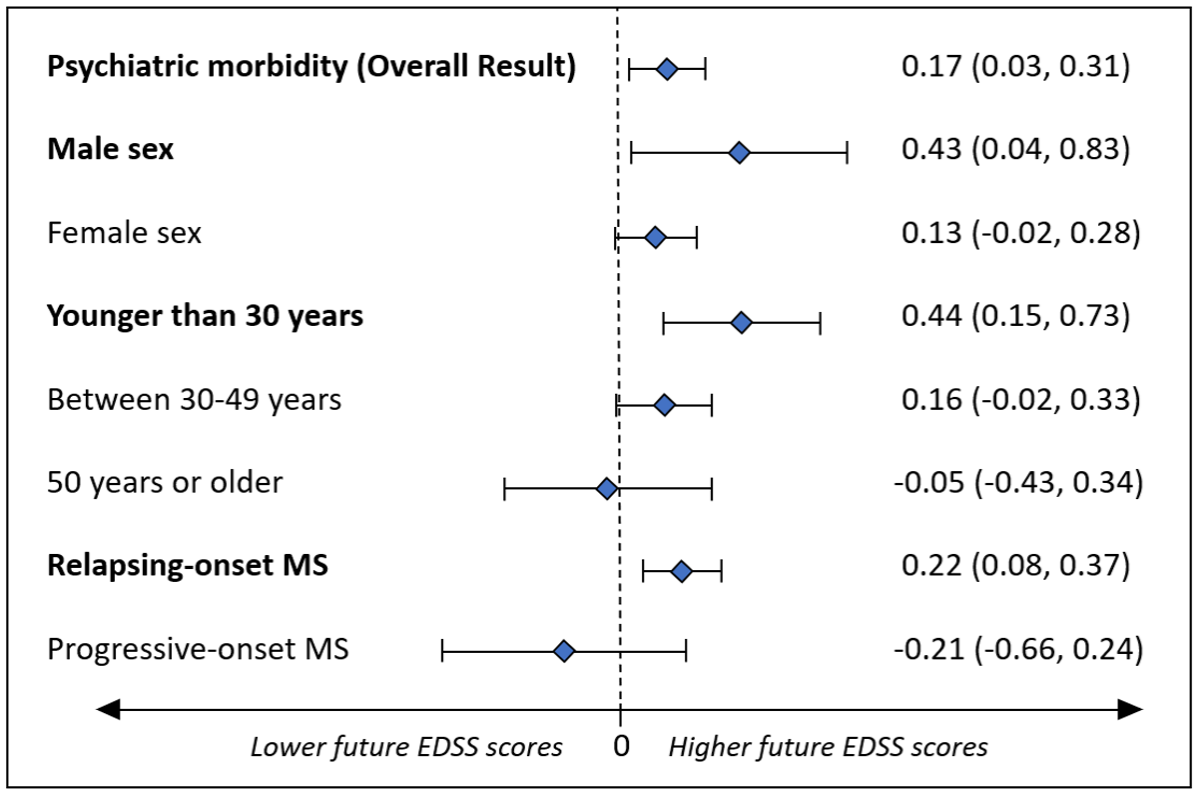
Effect of sex, age at MS onset, and MS course on the association between psychiatric morbidity pre-MS symptom onset and future neurological disability as measured by the Expanded Disability Status Scale (EDSS). Results represent adjusted β-coefficients with 95% confidence intervals.

Psychiatric morbidity during the 5 years before MS onset was significantly associated with higher subsequent EDSS scores (adjβ-coefficient = 0.17; 95% CI: 0.03–0.30) over follow-up. When examined for whether sex, age at MS onset, or MS course modified the association between psychiatric morbidity and future EDSS, the relationship was more pronounced for males (adjβ-coefficient = 0.43; 95% CI: 0.04–0.83; *p* = 0.03), younger individuals (<30 years; adjβ-coefficient = 0.44; 95% CI: 0.15–0.73; *p* < 0.01), and those with relapsing-onset MS (adjβ-coefficient = 0.22; 95% CI: 0.08–0.37; *p* < 0.01). Results were not significant for females, older persons, or those with progressive-onset MS, although some 95% CIs were wide (Figure 1). When baseline EDSS was added to the model, the association between psychiatric morbidity and subsequent EDSS was markedly attenuated (adjβ = 0.06; 95% CI: −0.04 to 0.15) while baseline EDSS was strongly predictive of later scores (adjβ = 0.72; 95% CI: 0.69–0.76). DMD exposure did not materially change the association between psychiatric morbidity and subsequent EDSS: uptake did not differ between groups (*p* = 0.21); removing DMD from the model changed β by 6%; and the psychiatric morbidity×DMD interaction was not significant (*p* = 0.71) (analyses not shown).

**Figure 2.**
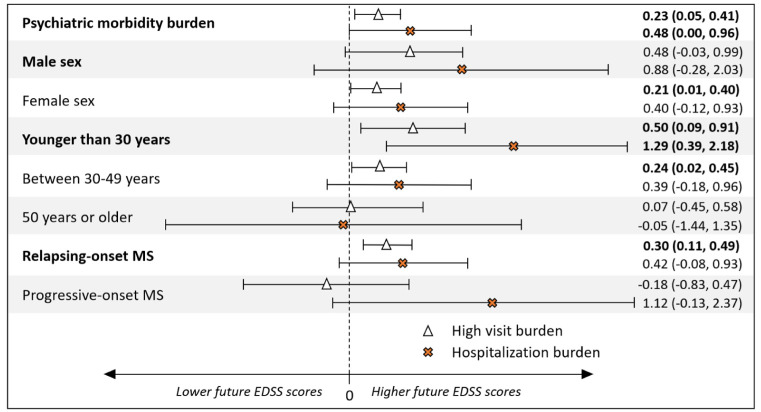
The burden of physician visit or hospitalization-related psychiatric morbidity in the 5 years before MS onset and future Expanded Disability Status Scale (EDSS) scores and the effect of sex, age at MS onset, and MS course. Results represent adjusted β-coefficients with 95% confidence intervals. Psychiatric morbidity burden was assessed in the 481 individuals who fulfilled the criteria for psychiatric morbidity as per Supplemental Table 1. Of these, 235/481 had a *high physician visit burden* (>median number of psychiatric-related physician visits) and 37/481 had ⩾1 psychiatric-related hospital admission (“*hospitalization burden*”). Individuals not meeting the criteria for psychiatric morbidity (*n* = 1731) formed the reference group.

In the complementary analysis, 235/481 (48.9%) individuals with psychiatric morbidity were classified as having a high psychiatric-related physician visit burden per its definition (see *complementary analyses*), while 37/481 (7.7%) had a hospitalization burden. Compared with those with no psychiatric morbidity, a high psychiatric-related physician visit burden in the 5-years pre-MS onset was associated with increased EDSS scores (adjβ-coefficient = 0.23; 95% CI: 0.05–0.41; *p* = 0.01). Similarly, having ⩾1 psychiatric-related hospitalization was also associated with higher EDSS scores (adjβ-coefficient = 0.48; 95% CI: 0.002–0.96; *p* = 0.049). Sex, age at MS onset, and MS course modified the association between psychiatric morbidity burden and future EDSS (Figure 2). For those with a high psychiatric-related physician visit burden, the relationship was more pronounced for females (adjβ-coefficient = 0.21; 95% CI: 0.01–0.40; *p* = 0.04), those younger at MS onset (⩽30 years: adjβ-coefficient = 0.50; 95% CI: 0.09–0.91; *p* = 0.02; 30–49 years: adjβ-coefficient = 0.24; 95% CI: 0.02–0.45; *p* = 0.03), and with relapsing-onset MS (adjβ-coefficient = 0.30; 95% CI: 0.11–0.49; *p* < 0.01). Findings were in a similar direction for those younger at MS onset (⩽30 years: adjβ-coefficient = 1.29; 95% CI: 0.39–2.18; *p* < 0.01) with hospitalization burden. Results were not statistically significant for males, older persons, or those with progressive-onset MS, although the 95% CIs were rather wide.

## Discussion

In this large cohort study linking MS-specific clinical records with population-based health administrative data, psychiatric morbidity before MS onset was associated with greater subsequent neurological disability. This association was particularly pronounced in males, individuals <30 years, and those with relapsing-onset MS. These findings suggest that psychiatric morbidity, even in the very early stages of the disease, can be associated with future neurological disability in MS, particularly in these specific subgroups.

We were unable to find another study exploring the association between psychiatric morbidity *pre-MS onset* and future disability outcomes. Previous studies examining this association *post-MS onset* have yielded mixed results.^15–18^ However, most either relied on self-reported data (e.g., patient-reported EDSS scores) or used cross-sectional designs, which may have contributed to variability in findings. Nonetheless, our results align with two longitudinal studies conducted in Canada and Sweden, and a meta-analyses using data from several phase 3 MS-DMD clinical trials. The Canadian study, which included 2312 pwMS, some who may overlap with our cohort due to shared data sources, found that mood and/or anxiety disorders, mostly measured *post-MS onset*, were significantly associated with higher future EDSS (adjβ coefficient = 0.28, *p* < 0.01).^6^ The Swedish study, involving 5875 pwMS, showed that those with depression had a significantly higher risk of reaching various EDSS-related disability milestones, including a subgroup diagnosed with depression in the 2-years pre-MS onset.^19^ This study focused exclusively on individuals with unipolar depression and relied solely on specialist care data. In contrast, our study offers a more comprehensive and representative picture of psychiatric morbidity in the MS population by examining a broader spectrum of conditions and incorporating data from all healthcare interactions, including primary care. Finally, a cohort study using a meta-analytic approach to assess comorbidities in >16,000 MS clinical trial participants found that the risk of disability worsening and disease relapse progressively increased with the number of psychiatric comorbidities.^20^ When specific conditions were analyzed, only depression was significantly associated with these outcomes, whereas anxiety and other psychiatric disorders were not independently linked to disability worsening or disease relapse.^20^ While that study provides further evidence of the association between psychiatric comorbidities and MS outcomes, it differs from ours in several aspects. Specifically, it assessed individuals only *post-MS diagnosis* and relied on clinical trial populations, potentially limiting generalizability of its findings to the broader MS population. In addition, comorbidity status across the different trials was based on medical records, which likely varied in accuracy. Information on the timing of comorbidity development was unavailable, limiting the ability to examine its temporal relationship with MS outcomes.

The association between psychiatric morbidity and future neurological disability may be influenced by several mechanisms. Inflammatory dysregulation, a core feature of early MS pathology, has been implicated in the development of psychiatric disorders^21^ and could contribute to increased neurodegeneration and subsequent disability. In addition, psychiatric morbidity may lead to maladaptive coping strategies and poor health behaviors, such as smoking,^22^ physical inactivity,^23^ or DMD non-adherence,^24^ potentially altering MS course. Conversely, psychiatric morbidity might develop in response to the experience of worsening disability.^25^ However, this seems unlikely for the psychiatric morbidity observed in our cohort, as we examined psychiatric conditions occurring pre-MS symptom onset, a disease stage where disability is likely minimal/absent. It remains possible, though, that some psychiatric morbidity arose as a consequence of being in the early stages of the disease, that is, in response to other nonspecific signs/symptoms often experienced in the MS prodrome.^3^

Finally, higher psychiatric morbidity and faster disability accumulation may, to some degree, be driven by differences in SES, as individuals with lower SES often experience greater exposure to chronic stress, reduced healthcare access, and higher comorbidity burden. Lower SES is also associated with maladaptive coping behaviors, such as lower treatment adherence or unhealthy lifestyle choices, which may further influence disease progression.^26^ Although our analyses adjusted for SES, residual confounding is possible, as neighborhood-based income measures may not fully capture individual socioeconomic disparities.^27^

We found that the association between psychiatric morbidity pre-MS onset and future disability was stronger in male pwMS. Previous studies on psychiatric morbidity occurring *after* MS onset have shown inconsistent results, with some reporting significant associations only in females.^6^ Our findings suggest that sex may interact with disability differently when psychiatric conditions develop *before* MS onset. However, the wide CIs for males indicate variability, underscoring the need for larger cohorts to confirm these differences. We also observed a more pronounced association in younger individuals. Although reasons are not entirely clear, contributing factors could include less effective coping mechanisms,^28^ age-specific stressors,^29^ higher frequency of unhealthy behaviors,^30^ more inflammatory MS activity,^31^ and differential impact of treatment decisions across younger pwMS,^32^ among others. Another possible explanation is that in younger individuals, who generally have lower disability and shorter disease duration, the disease trajectory may be more easily altered, making changes in disability more noticeable. Finally, the association appeared stronger in individuals with relapsing-onset MS, perhaps reflecting the higher inflammatory activity characteristic of early disease; psychiatric symptoms are closely linked to such activity and can even precede clinical relapses/radiological disease activity.^21^ The influence of psychiatric conditions may also diminish in the face of advanced neurodegenerative changes, perhaps accelerated by aging. This would align with the weaker association observed in progressive-onset MS and older subjects. However, the smaller sample size for progressive-onset MS might have limited our ability to detect associations. In addition, interpreting findings in this subgroup can be challenging due to difficulties in assigning them a clear symptom onset.

Our complementary analysis, examining the relationship between psychiatric morbidity and future EDSS in subgroups of individuals with high psychiatric-related physician visit burden and psychiatric hospitalizations pre-MS symptom onset, revealed a more pronounced association when these specific burdens were considered, particularly for psychiatric hospitalizations. For instance, individuals <30 years with psychiatric morbidity and ⩾1 psychiatric hospitalization in the 5 years before MS onset showed a substantially higher estimated increase in their EDSS (adjβ coefficient = 1.29, *p* < 0.01) compared with those without psychiatric morbidity. Although nonsignificant, results were on a similar direction for individuals with ⩾1 psychiatric hospitalization and primary progressive onset. The stronger association between psychiatric morbidity and future MS disability outcomes when these healthcare use-related burdens were considered might be explained by several potential mechanisms. For instance, increased psychiatric-related healthcare use, particularly hospitalizations, could reflect greater severity of psychiatric illness, which may in turn negatively impact MS outcomes. High visit frequency also likely captures downstream care needs (e.g., medication adjustments, crisis follow-ups, and safety assessments) reinforcing its role as an indicator of symptom load and treatment complexity. Alternatively, a more active early/prodromal MS phase might lead to increased psychiatric morbidity, resulting in higher utilization of psychiatric healthcare services. It is also possible that both mechanisms contribute partially to this association and reinforce one another. Studies allowing to access a diverse range of health-related behaviors before MS onset will be necessary to clarify the complex interplay between psychiatric-related healthcare use and MS disability.

Our study has several limitations. First, the associations observed may be confounded by unmeasured factors, including differential access to care or underlying health behaviors not captured in the data. Second, administrative data cannot fully capture self-managed symptoms or encounters with non-physician providers (e.g., counselors and therapists). In addition, the study lacked details on the severity of mental health symptoms, treatment adherence, or quality of care, which could influence the observed relationships. We also did not have MRI data; however, as early prodromal MS symptoms are frequently nonspecific, they may not trigger imaging studies during this phase of the disease. It is possible that neurologists may occasionally misdate first MS symptoms, so a small fraction of psychiatric encounters could occur after rather than before MS clinical onset, but this should affect only a minority of cases and is unlikely to alter our results. Post hoc adjustments suggest that the excess disability in individuals with psychiatric morbidity pre-MS onset is largely explained by their higher disability at baseline rather than by accelerated accumulation thereafter. Finally, frequency of psychiatric morbidity may be underestimated due to the low sensitivity of administrative data, care avoidance due to stigma, and physician under-coding. These limitations warrant cautious interpretation of the findings and underscore priorities for future research.

Study strengths include the use of a large, representative cohort of MS-clinic attendees over nearly three decades, and the prospective collection of data, with exposures and outcomes recorded independently, minimizing recall and related biases. The broad inclusion criteria reduced selection bias, while the application of a validated algorithm, using information from virtually every health encounter in the province, ensured accurate identification of psychiatric morbidity. These methodological strengths contribute to the robustness of our findings.

## Conclusion

Psychiatric morbidity is highly prevalent in MS and was associated with greater subsequent neurological disability even when present before MS onset. Males, younger individuals, and those with relapsing-onset MS were more predisposed to increased future disability in the presence of psychiatric morbidity in the 5 years before onset of MS. These associations were stronger in individuals with a high burden of psychiatric-related visits and psychiatric hospitalizations.

Psychiatric conditions often remain underdiagnosed and undertreated in MS,^33,34^ despite availability of effective treatments.^35^ Given that psychiatric conditions can appear very early in MS and are associated with future neurological disability, they likely need to be assessed and managed at the time of MS diagnosis, or even earlier if possible. Future research should explore whether promptly addressing psychiatric morbidity with appropriate interventions, such as counseling, psychotherapy, or medication, can potentially mitigate disability accumulation in MS.

## Supplemental Material

sj-docx-1-msj-10.1177_13524585251382801 – Supplemental material for Psychiatric morbidity during the multiple sclerosis prodrome is associated with future disability Supplemental material, sj-docx-1-msj-10.1177_13524585251382801 for Psychiatric morbidity during the multiple sclerosis prodrome is associated with future disability by Anibal S Chertcoff, Marta Ruiz-Algueró, Fardowsa Yusuf, Yinshan Zhao, Feng Zhu, Ruth Ann Marrie and Helen Tremlett in Multiple Sclerosis Journal

sj-tif-2-msj-10.1177_13524585251382801 – Supplemental material for Psychiatric morbidity during the multiple sclerosis prodrome is associated with future disability Supplemental material, sj-tif-2-msj-10.1177_13524585251382801 for Psychiatric morbidity during the multiple sclerosis prodrome is associated with future disability by Anibal S Chertcoff, Marta Ruiz-Algueró, Fardowsa Yusuf, Yinshan Zhao, Feng Zhu, Ruth Ann Marrie and Helen Tremlett in Multiple Sclerosis Journal
