# Supplementary material for: Psychiatric morbidity during the multiple sclerosis prodrome is associated with future disability
Source: Mult Scler. 2025 Oct 26;31(14):1619–28. doi: 10.1177/13524585251382801 (PMC12644253; doi:10.1177/13524585251382801)
Supplement: sj-docx-1-msj-10.1177_13524585251382801 – Supplemental material for Psychiatric morbidity during the multiple sclerosis prodrome is associated with future disability [file sj-docx-1-msj-10.1177_13524585251382801.docx]

**Supplementary Table 1. Diagnostic codes and algorithm used to identify psychiatric morbidity using administrative health data. Modified from Marrie et al**^9^

| **Morbidity** | **Algorithm** | **ICD-9 codes** | **ICD-10-CA codes** | **BC diagnostic codes**^*^ |
| --- | --- | --- | --- | --- |
| Psychiatric morbidity | ≥5P and/or ≥1H^‡^ within a 5-year period (with ≥1 visit occurring before MS onset) | 295.x, 296.0, 296.04, 296.1, 296.14, 296.2, 296.3, 296.4, 296.44, 296.5, 296.54, 296.6, 296.7, 296.8, 298.0, 300.0, 300.2, 300.4, 311 | F20.x, F25, F31, F32, F33, F34, F40, F41 | 50B |

ICD: International Classification of Disease; P: Physician visits; H: Hospital admissions. ^*^BC diagnostic codes: additional physician codes specific to British Columbia, Canada. Code 50B is used for both depression and anxiety.^7^ ^‡^To avoid double counting, hospitalizations occurring within one day (24 hours) of the previous hospitalization or overlapping hospitalizations (transfers) were considered the same hospitalization and counted once.

**Supplementary Figure 1. Timelines for assessment of the study exposure and outcome.**

Assessment of psychiatric morbidity (exposure) was examined from 5 years before to MS symptom onset using psychiatric-related physician visits and hospital admissions. EDSS scores (outcome) were obtained from neurological assessments recorded at a routine MS clinic visit (individual Functional System sub-scores were not assessed as they were not available). To fulfill the psychiatric case definition, participants had ≥5 physician visits and/or 1 hospital admission for psychiatric disease within 5 years, with ≥1 physician or hospital visit occurring within the 5 years pre-MS symptom onset. The figure depicts one scenario in which all qualifying visits happened to occur before MS onset. The mean (SD) time from MS symptom onset to the first EDSS assessment was 3.2 (3.3) years. The mean (SD) time from first to last EDSS assessment was 5.2 (4.9) years.
