# Supplementary figures and images for: Psychiatric morbidity during the multiple sclerosis prodrome is associated with future disability
Source: Mult Scler. 2025 Oct 26;31(14):1619–28. doi: 10.1177/13524585251382801 (PMC12644253; doi:10.1177/13524585251382801)

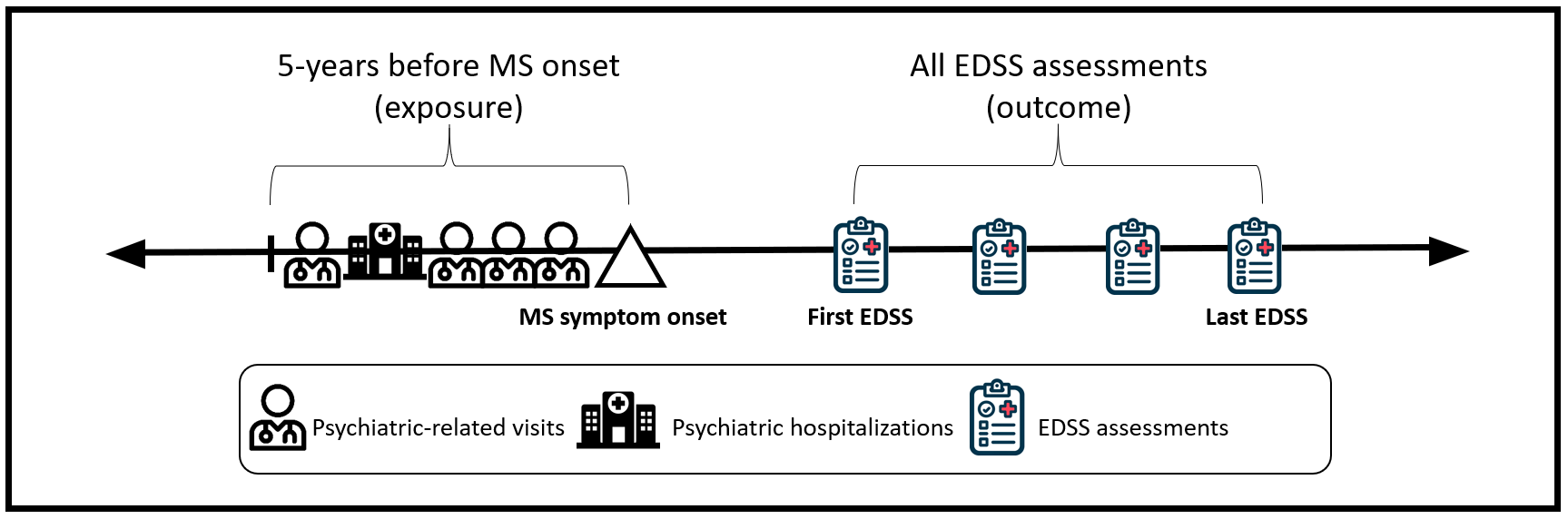

Supplement: sj-tif-2-msj-10.1177_13524585251382801 – Supplemental material for Psychiatric morbidity during the multiple sclerosis prodrome is associated with future disability [file sj-tif-2-msj-10.1177_13524585251382801.tif]
